# Clinical, genomic, and functional characterization of vancomycin-resistant *Enterococci* from immunocompromised patients: insights into epithelial dysfunction and bloodstream infections

**DOI:** 10.3389/fcimb.2025.1726074

**Published:** 2025-12-16

**Authors:** Giuseppe Sangiorgio, Ilenia Martina Pia Filannino, Giuseppe Migliorisi, Dafne Samantha Irene Bongiorno, Nicolò Musso, Grete Francesca Privitera, Gabriella Santuccio, Dario Leotta, Maddalena Calvo, Stefania Stefani

**Affiliations:** 1 Department of Biomedical and Biotechnological Sciences, University of Catania, Catania, Italy; 2 U.O.C. Laboratory Analysis Unit, A.O. “G.F. Ingrassia”, Palermo, Italy; 3 Advanced and Innovative Diagnostic Academy (A.I.D.A.) S.r.l., Spin-off of BRIT Research Center, University of Catania, Catania, Italy; 4 Department of Medicine and Surgery, University of Enna “Kore”, Enna, Italy; 5 Department of Clinical and Experimental Medicine, University of Catania, Catania, Italy; 6 Complex Operational Unit of Hematology, A.O.U. Policlinico “G. Rodolico-San Marco”, Catania, Italy; 7 U.O.C. Laboratory Analysis II, A.O.U. Policlinico “G. Rodolico- San Marco”, Catania, Italy

**Keywords:** host-pathogen interaction, *Enterococcus* spp., immunocompromised host, whole genome sequencing, molecular characterization

## Abstract

**Background:**

Vancomycin-resistant *Enterococcus faecium* and *Enterococcus faecalis* (VRE) are increasingly recognized as major opportunistic pathogens in immunocompromised patients, where they may cause bloodstream infections (BSIs). The present study aimed to characterize a cohort of immunocompromised patients colonized or infected with VRE, performing genomic analysis of these isolates. Additionally, we investigated the impact of bacterial culture supernatants on Caco-2 epithelial cells, focusing on adhesion and cytotoxicity to elucidate mechanisms related to epithelial dysfunction and bacterial translocation.

**Methods:**

We conducted a retrospective study including 46 VRE from two Italian hospitals. Clinical and epidemiological data were collected, and isolates were characterized by antimicrobial susceptibility testing and whole-genome sequencing. Four representative isolates (*E. faecium* ST80, *E. faecium* ST117, *E. faecalis* ST28, and *E. faecalis* ST179) and two reference strains (ATCC 29212™ and ATCC 51299™) were selected for *in vitro* analyses. Adhesion to Caco-2 monolayers was quantified, while cytotoxicity was assessed using MTT assays with bacterial cell-free supernatants (CS). Hydrogen peroxide (H_2_O_2_) production was measured using the Amplex^®^ Red Hydrogen Peroxide/Peroxidase Assay Kit.

**Results:**

The majority of isolates were *E. faecium* (78.3%), predominantly ST80 and ST117, possessed multiple resistance determinants. *E. faecalis* isolates displayed greater sequence type diversity with a ST28 predominance, carrying virulence genes as *ebp*, *gelE*, and *elrA*. *In vitro*, bloodstream-derived isolates (*E. faecium* 51, *E. faecalis* 52) and reference strain ATCC 29212™ adhered more strongly to Caco-2 cells than other isolates. CS from invasive isolates and ATCC 51299™ significantly reduced epithelial cell viability at 24 h (p < 0.01). In these isolates, H_2_O_2_ higher quantification was documented in a cellular model.

**Discussion:**

Our findings highlighted the convergence of antimicrobial resistance and virulence traits in VRE, alongside functional evidence of strain-dependent adhesion and secretion of cytotoxic metabolites. Elevated H_2_O_2_ production provides a possible path between enterococcal secretomes and epithelial injury, suggesting oxidative stress as a contributor to epithelial dysfunction and potential translocation. These insights expand current understanding of VRE pathogenesis and point to novel therapeutic approaches aimed at preserving epithelial integrity and mitigating oxidative damage in high-risk patients.

## Introduction

*Enterococcus faecium* and *Enterococcus faecalis* resistant to vancomycin (VRE) have emerged as major opportunistic pathogens. The World Health Organization described these species as a global public health threat (WHO, 2017; Organization WHO, 2022). In hospital settings, VRE readily colonizes the intestines of vulnerable patients, revealing a rapid outgrowth that predisposes to self-contamination during routine procedures. Moreover, critical patients often underwent antibiotic treatments, chemotherapy, surgery, neutropenia, and other risk factors compromising the intestinal barrier integrity. Once the damage has occurred, *Enterococcus* spp. migrates across the intestinal epithelium, disseminating and causing bacteremia episodes (Boucher et al., 2009; Chilambi et al., 2020). *Enterococci* constitute less than 0.1% of the healthy intestinal microbiota. However, their abundance can dramatically increase in immunocompromised and hospitalized patients (Ubeda et al., 2010; Sangiorgio et al., 2024a). Extensive molecular epidemiological and genomic studies have shown that hospital-associated *E. faecium* strains belong to a distinct phylogenetic lineage, initially designated clonal complex-17 and later reclassified as clade A1 (Lebreton et al., 2013; Arredondo-Alonso et al., 2020). Clade A1 strains harbor unique genetic determinants, including putative virulence factors, genes encoding antibiotic transport and intestinal colonization proteins, and carbohydrate metabolism genes that support the utilization of host-derived amino sugars (Archambaud et al., 2019). These adaptations contribute to extensive colonization of the dysbiotic intestine, a common factor among hospitalized patients receiving prolonged antibiotic treatment (Zhang et al., 2013; Archambaud et al., 2019). Furthermore, the antibiotic-driven dysbiosis alters intestinal architecture, including thinning of the mucus layer, disruption of epithelial junctions, and deformation of E-cadherin–mediated adhesion. In murine colonization models, enterococcal outgrowth has been associated with mucus layer depletion and aberrant trapping of *E. faecium* clade A1 in extracellular matrix components (Hendrickx et al., 2015).

Despite these considerations, there is limited current knowledge about VRE soluble factors secretion and the human intestinal epithelium. Bacterial culture supernatants may contain metabolites, enzymes, peptides, and virulence-associated molecules. All of these elements influence epithelial viability, barrier integrity, and immune signaling. The Caco-2 cell line provides a suitable platform to investigate similar interactions due to its well-known usage as an intestinal epithelium *in vitro* model.

Herein, we present a clinical and epidemiological description of immunocompromised patients colonized or infected with VRE. We propose a molecular and microbiological characterization of the involved enterococcal isolates. We investigated *Enterococcus* spp. strains’ culture supernatants effects on Caco-2 cells. Specifically, we assessed adhesion and cytotoxicity to understand the mechanisms underlying intestinal epithelial dysfunction. Our *in vivo* and *in vitro* patient-derived data aim to comprehensively clarify VRE pathogenesis and potential virulence markers in translocation and infection processes.

## Results

### Clinical and epidemiological characterization

The study included 46 patients, with a mean age of 59.7 years (SD = 15.05 years). The cohort comprised 52.9% males, and all individuals were immunocompromised. Most patients were hospitalized in the hematology ward (52.2%), with smaller proportions in internal medicine (19.6%), oncology (15.2%), neurology (8.7%), and pulmonology (4.3%) units. For immunocompromised patients, both hospitals reported higher VRE incidences compared to other patient categories. At the University Policlinico of Catania, among hematological patients, VRE was isolated in 30.5% of intestinal samples and 7.8% of blood cultures; among other patient categories in this hospital, the rates were 12.4% (intestinal) and 2.6% (blood). At Civico Hospital of Palermo, immunocompromised patients had 33.4% VRE isolation from intestinal samples and 30.2% from blood cultures, while other patient categories had 22.8% (intestinal) and 18.7% (blood).

Most patients had an underlying neoplastic malignancy (91.3%), while diabetes mellitus was present in 23.9%. Based on the age-adjusted Charlson Comorbidity Index, 69.6% of patients were classified as high risk (score ≥5), and 30.4% as moderate risk (score 2–4). Bloodstream infections were documented in 41.3% of cases, and co-infections were identified in 13.3%. Interestingly, all the documented bloodstream infection episodes were acquired during the prolonged hospitalization of the immunocompromised patients. In total, 46 vancomycin-resistant *Enterococcus* spp. isolates were recovered. Species distribution revealed a predominance of *E. faecium* (78.3%) over *E. faecalis* (21.7%). The most frequent site of isolation was blood (41.3%), followed by rectal swabs (23.9%), stool samples (19.6%), and urine (15.2%). A detailed description of the demographics of the study population is shown in **Table 1**. Furthermore, **Table 2** shows the phenotypic susceptibility testing details.

**Table 1:** Demographic and clinical characteristics of the study population.

	Counts	Mean	SD
Age	46	59.7	15.05
Gender	Counts	% of Total	
F	23	50.0 %	
M	23	50.0 %	
Ward	Counts	% of Total	
Internal Medicine	9	19.6 %	
Neurology	4	8.7 %	
Hematology	24	52.2 %	
Pulmonology	2	4.3 %	
Oncology	7	15.2 %	
BSI	Counts	% of Total	
No	27	58.7 %	
Yes	19	41.3 %	
Site	Counts	% of Total	
Urine	7	15.2 %	
Coprocolture	9	19.6 %	
Rectal swab	11	23.9 %	
Blood	19	41.3 %	
Species	Counts	% of Total	
*E. faecalis*	10	21.7 %	
*E. faecium*	36	78.3 %	
Cancer	Counts	% of Total	
No	4	8.7 %	
Yes	42	91.3 %	
Diabetes	Counts	% of Total	
No	35	76.1 %	
Yes	11	23.9 %	
Charlson Comorbidity Index (age-adjusted)	Counts	% of Total	
Moderate (2-4)	14	30.4 %	
High (≥ 5)	32	69.6 %	
Co-infection	Counts	% of Total	
No	26	86.7 %	
Yes	4	13.3 %	

**Table 2:** Phenotypic antimicrobial susceptibility testing MIC ranges for the included strains.

Species	AMC	AMP	AMS	Q/D	CIP	LEV	LNZ	TEC	VAN	TGC
*E. faecium*	>16	>16	0.5-16	0.25-1	>4	>4	1-4	>16	>16	0.12-0.5
*E. faecalis*	2-16	2-16	2	/	4	0.5-4	1-2	>16	>16	0.12

Antibiotic tested: AUG, amoxicillin/clavulanic acid; AMP, ampicillin; AMS, ampicillin/sulbactam; QDA, quinupristin/dalfopristin; CIP, ciprofloxacin; LEV, levofloxacin; LNZ, linezolid; TEC, teicoplanin; VA, vancomycin; TGC, tigecycline.

**Figure 1:**
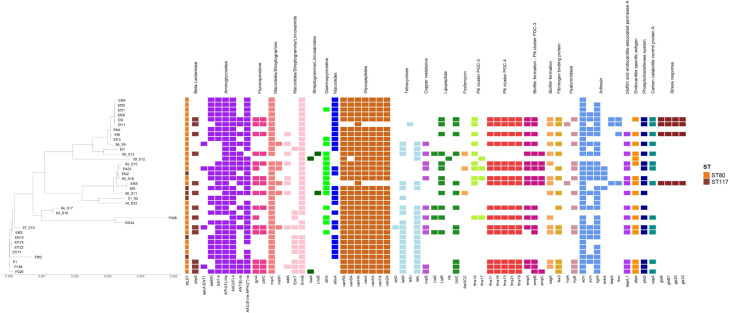
Phylogenetic relatedness and genomic features of *Enterococcus faecium*.

**Figure 2:**
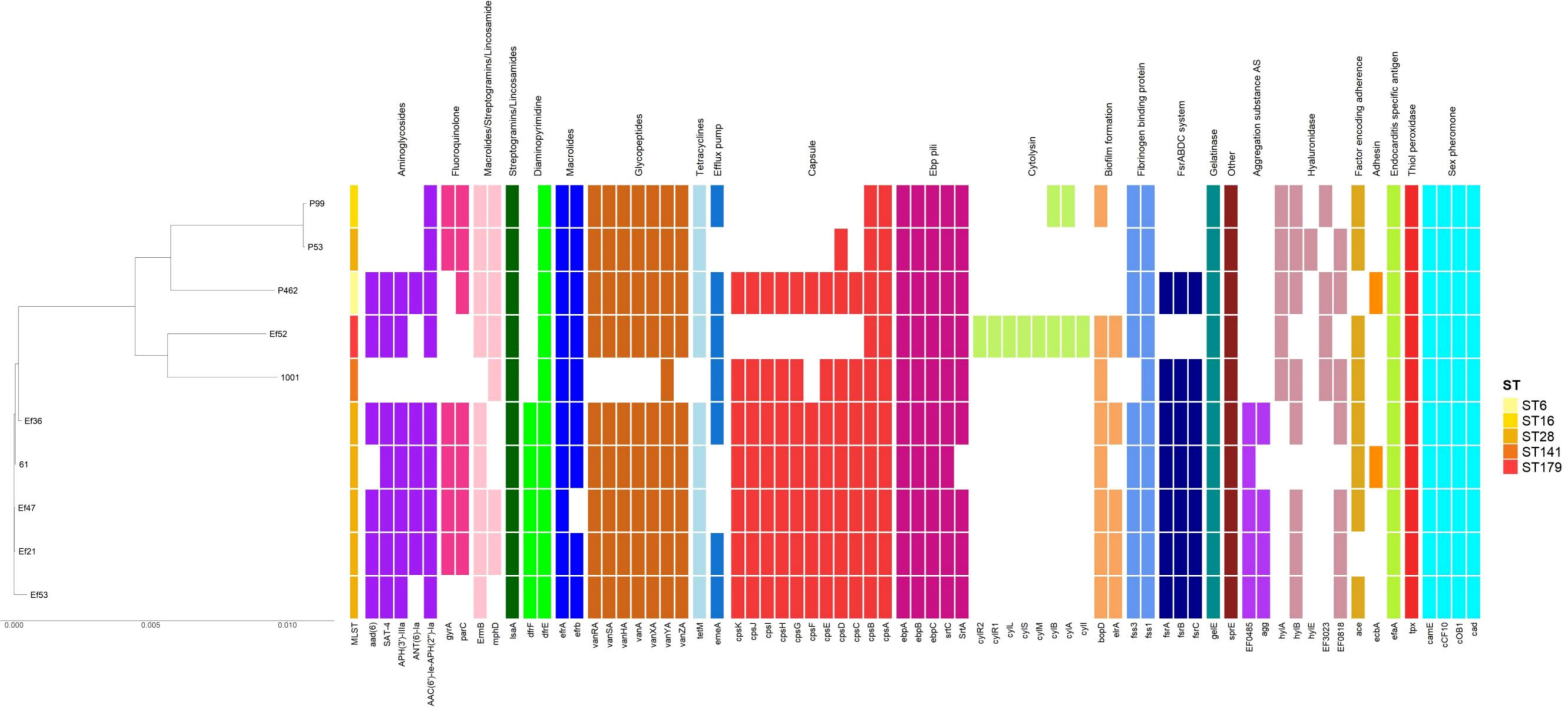
Phylogenetic relatedness and genomic features of *Enterococcus faecalis*.

**Figures 1**, **2** summarize the genomic analysis findings for *E. faecium* and *E. faecalis* isolates, while the corresponding data and the generated sequences are available in an online repository. The genomic analysis registered two different STs (ST80 and ST117) for *E. faecium*. Precisely, 30 strains (83.3%) belonged to ST80, whose resistance pattern included the *van*A operon’s elements (*van*A, *van*H*, van*R/*van*S*, van*X*, van*Y, and *van*Z), along with other resistance genes. The virulome analysis demonstrated the presence of *acm* (cell-wall anchored collagen adhesin) or *scm* (second collagen adhesin) genes in 28 (93.3%) *E. faecium* strains. Furthermore, 16 strains (53.3%) reported *emp* genes (*emp*A, *emp*B, and *emp*C), which correspond to pili formation, biofilm production, and extracellular adhesion episodes. Three ST80 isolates (10%) showed the *gls* protein-coding gene, related to bile salt tolerance and intestinal adaptation (Segawa et al., 2024; Dyrkell et al., 2021; Pratama et al., 2022; Rodríguez-Lucas et al., 2022). The remaining 6 *E. faecium* strains (20%) belonged to ST117, documenting *acm* or *scm* genes for adhesin production (Top et al., 2008; Chopjitt et al., 2024).

For *E. faecalis*, whole-genome sequencing (WGS) analysis found 5 different STs (ST28, ST179, ST141, ST6, and ST16). Of these, 6 isolates (60%) were ST28 and documented *van*A operon components. All ST28 E. faecalis carried the *gel*E gelatinase gene for bacterial invasion and motility (Waters et al., 2003; Creti et al., 2006; Thurlow et al., 2009; Montealegre et al., 2015; He et al., 2018; Pöntinen et al., 2021; El Zowalaty et al., 2023; Gore et al., 2025). One isolate (10%) was ST179 and harbored *efa*A (*Enterococcus faecalis* antigen A) (Coburn and Gilmore, 2003; Low et al., 2003; Waters et al., 2003; Thurlow et al., 2009; Kafil et al., 2016; He et al., 2018; Pöntinen et al., 2021; Șchiopu et al., 2023; El Zowalaty et al., 2023; Gore et al., 2025). One *E. faecalis* (10%) belonged to ST141 and showed adhesin-producing *ace* and *ebp* genes (Lebreton et al., 2009; Thurlow et al., 2009; Pöntinen et al., 2021; El Zowalaty et al., 2023). There was one ST6 (10%) with *bop*D, *ebp*, and *ace* genes in the virulence profile (Thurlow et al., 2009; Pöntinen et al., 2021; Șchiopu et al., 2023; El Zowalaty et al., 2023; Mirzaii et al., 2023). The analysis also revealed one ST16 (10%), which had a virulome pattern similar to the identified ST6 strain (Thurlow et al., 2009; Pöntinen et al., 2021; Șchiopu et al., 2023; El Zowalaty et al., 2023; Mirzaii et al., 2023).

### *In vitro* cell model experiments

To complement the epidemiological and clinical characterization of the patient cohort, a subset of isolates was selected for *in vitro* assays. Four representative *Enterococcus* strains were randomly chosen, each belonging to one of the most prevalent sequence types (ST80, ST117, ST28, and ST179). In addition, two well-characterized pathogenic reference strains were included: *E. faecalis* ATCC 29212™ and ATCC 51299™. The rationale for this selection was to ensure inclusion of both clinically relevant circulating clones and standard laboratory strains with established pathogenicity profiles, thereby providing a comparative framework for functional analyses. A detailed overview of the clinical characteristics of the patients from whom the isolates were obtained, as well as the phenotypic antimicrobial susceptibility profiles of the strains, is provided in **Supplementary Tables 1, 2**.

**Figure 3:**
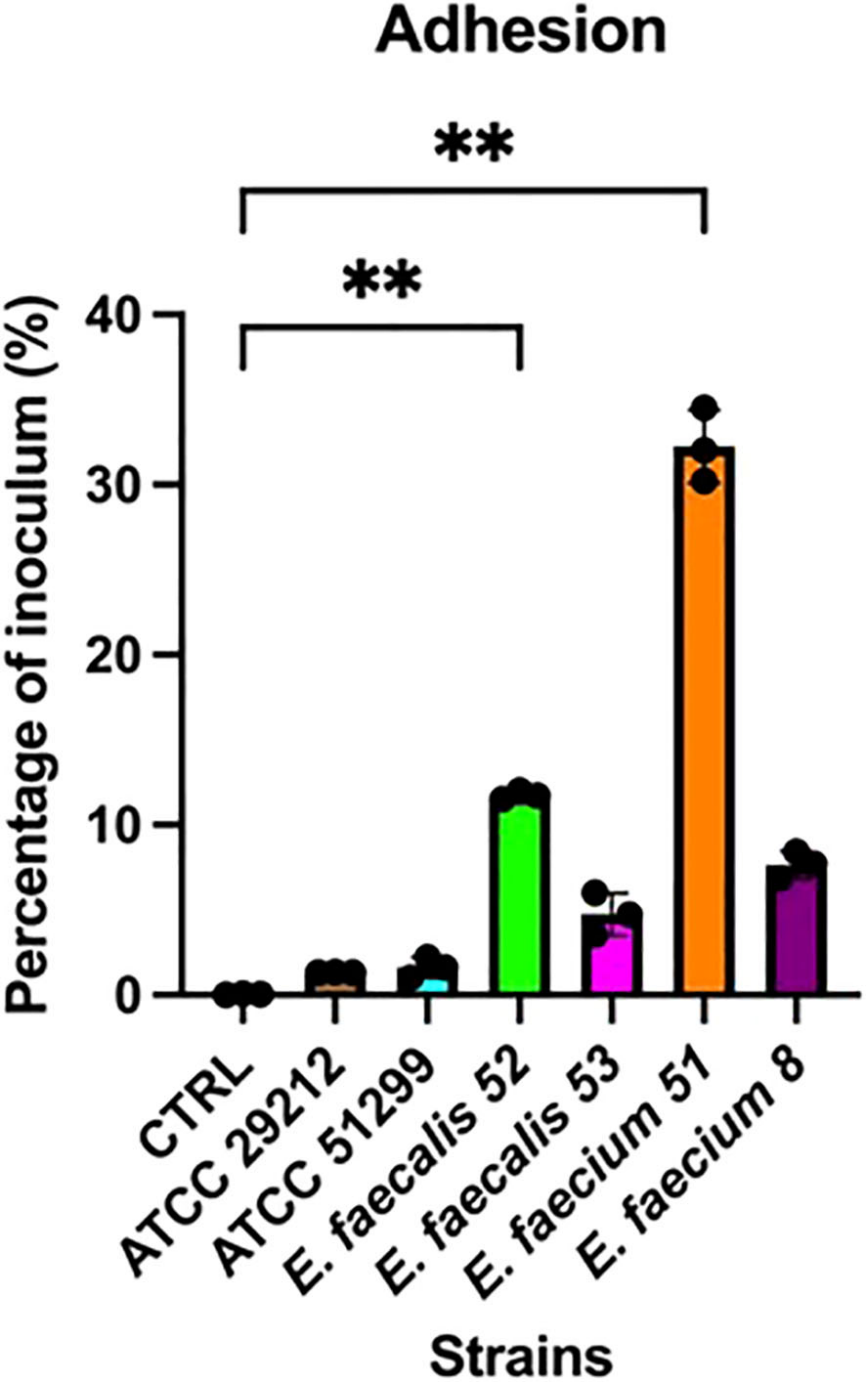
Adhesion capacity of reference isolates to epithelial cells. Each point represents an independent experiment (n = 3), performed with three technical replicates. Error bars indicate the standard deviation (SD). Statistical analysis was performed using one-way ANOVA followed by Tukey's post hoc test; *p*<0.05 was considered statistically significant (**p<0.01).

**Figure 4:**
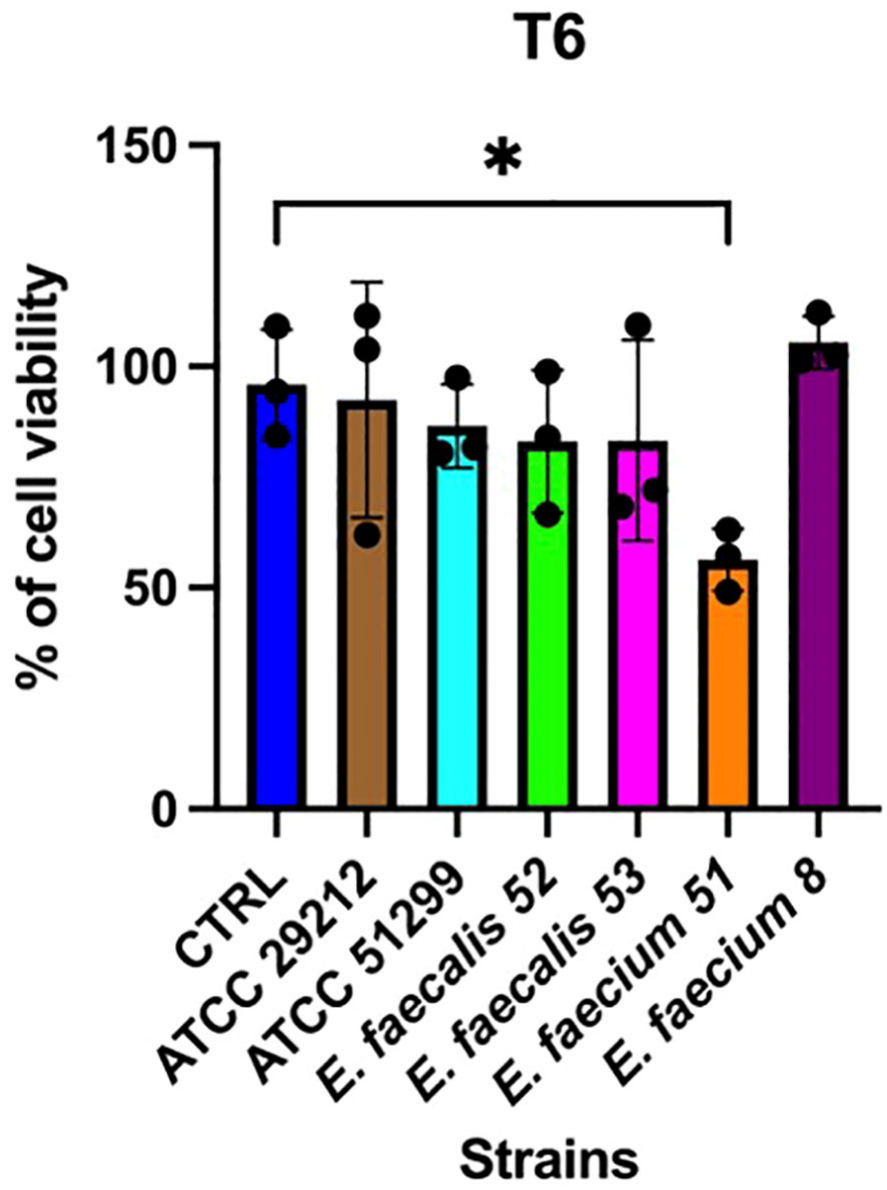
Effects of bacterial cell-free supernatants (CS) on Caco-2 cell viability at 6 h. Each point represents an independent experiment (n = 3), performed with three technical replicates. Error bars indicate the standard deviation (SD). Statistical analysis was performed using one-way ANOVA followed by Tukey's post hoc test; *p*<0.05 was considered statistically significant (*p<0.05).

**Figure 5:**
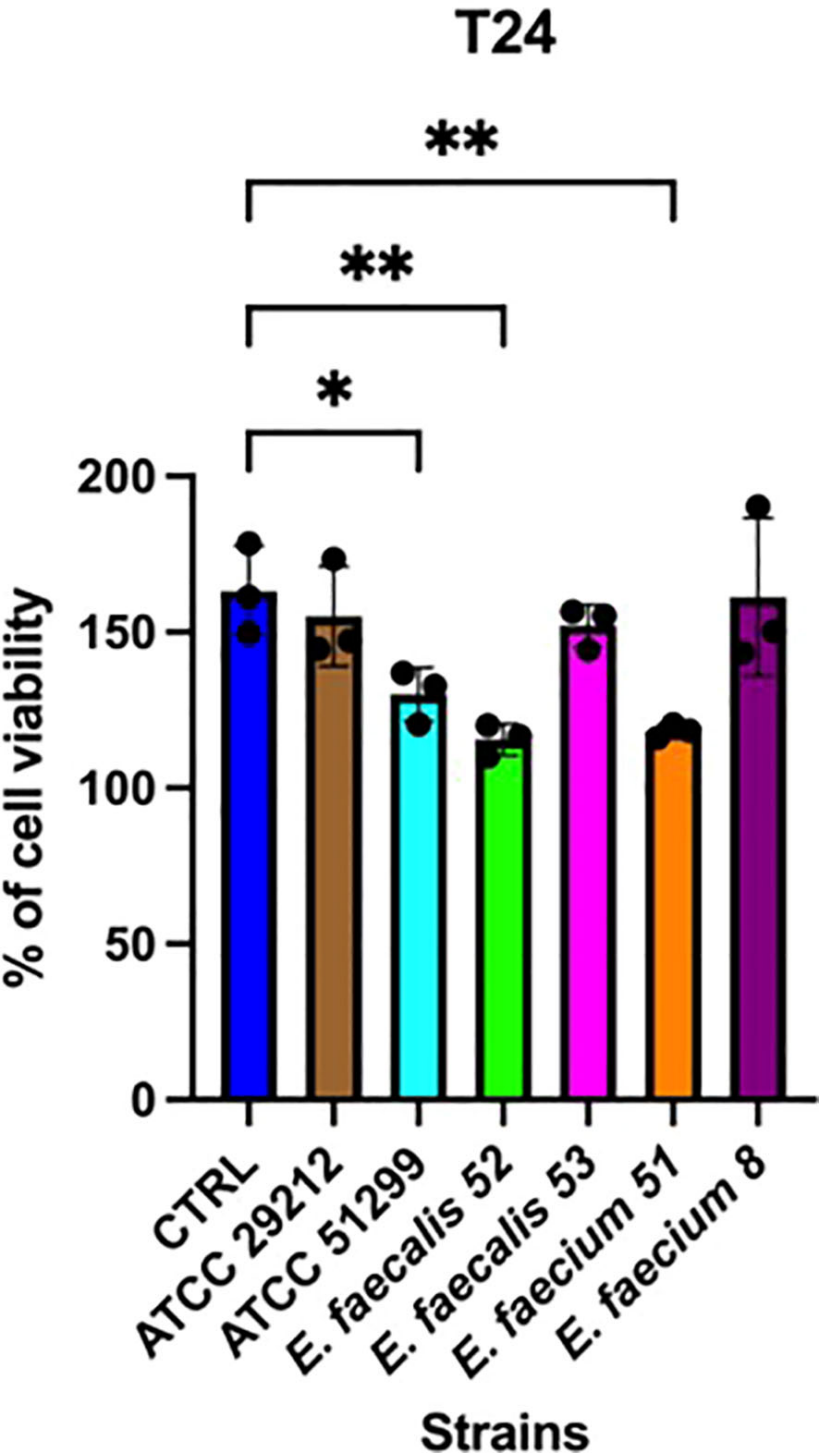
Effects of bacterial cell-free supernatants (CS) on Caco-2 cells after 24 h of exposure. Each point represents an independent experiment (n = 3), each performed with three technical replicates. Error bars indicate the standard deviation (SD). Statistical analysis was performed using one-way ANOVA followed by Tukey's post hoc test; p < 0.05 was considered statistically significant (*p < 0.05; **p < 0.01).

Adhesion assays revealed marked differences in adherence capacity among the tested strains. Notably, isolates *E. faecium* isolate 51 and *E. faecalis* isolate 52 consistently exhibited higher adherence than the other clinical strains (*p* < 0.01), consistent with their bloodstream infection origin, and significantly exceeded the levels observed in the reference isolates (**Figure 3**). The cytotoxicity assay revealed distinct strain-dependent effects on epithelial cell viability. Overall, exposure to bacterial CS resulted in reduced Caco-2 cell growth compared with controls. At the first time point (6 h), a significant decrease was observed for *E. faecium* isolate 51 (*p* < 0.05), whereas CS from isolate 8 induced a slight but non-significant increase in cell growth (**Figure 4**). By the second time point (24 h), the inhibitory effects were more pronounced (**Figure 5**). Caco-2 viability was significantly reduced following treatment with CS from *E. faecium* isolate 51 and *E. faecalis* isolate 52 (*p* < 0.01), as well as from the multidrug-resistant reference strain ATCC 51299™ (*p* < 0.05). No significant differences were detected for the remaining clinical isolates or for the susceptible reference strain.

Finally, given the cytotoxic effects observed with certain supernatants, we investigated whether *Enterococcus* isolates could produce metabolites capable of damaging the colonic epithelium. Several *Enterococcus* species are known to generate reactive oxygen species, including hydrogen peroxide (H_2_O_2_), which can impair epithelial integrity (Huycke et al., 2002). Genes such as *sod*A, encoding superoxide dismutase, have been implicated in oxidative stress responses and may contribute to this phenotype (Krishnamurthy et al., 2024). To explore this possibility, we adapted the Amplex^®^ Red Hydrogen Peroxide/Peroxidase Assay Kit, commonly used to quantify H_2_O_2_ release from mammalian leukocytes during oxidative bursts, to measure H_2_O_2_ output from Caco-2 cells exposed to bacterial CS. The assay revealed a significant increase in extracellular H_2_O_2_ levels following exposure to CS from *E. faecium* isolate 51 and *E. faecalis* isolate 52 (p < 0.01).

**Figure 6:**
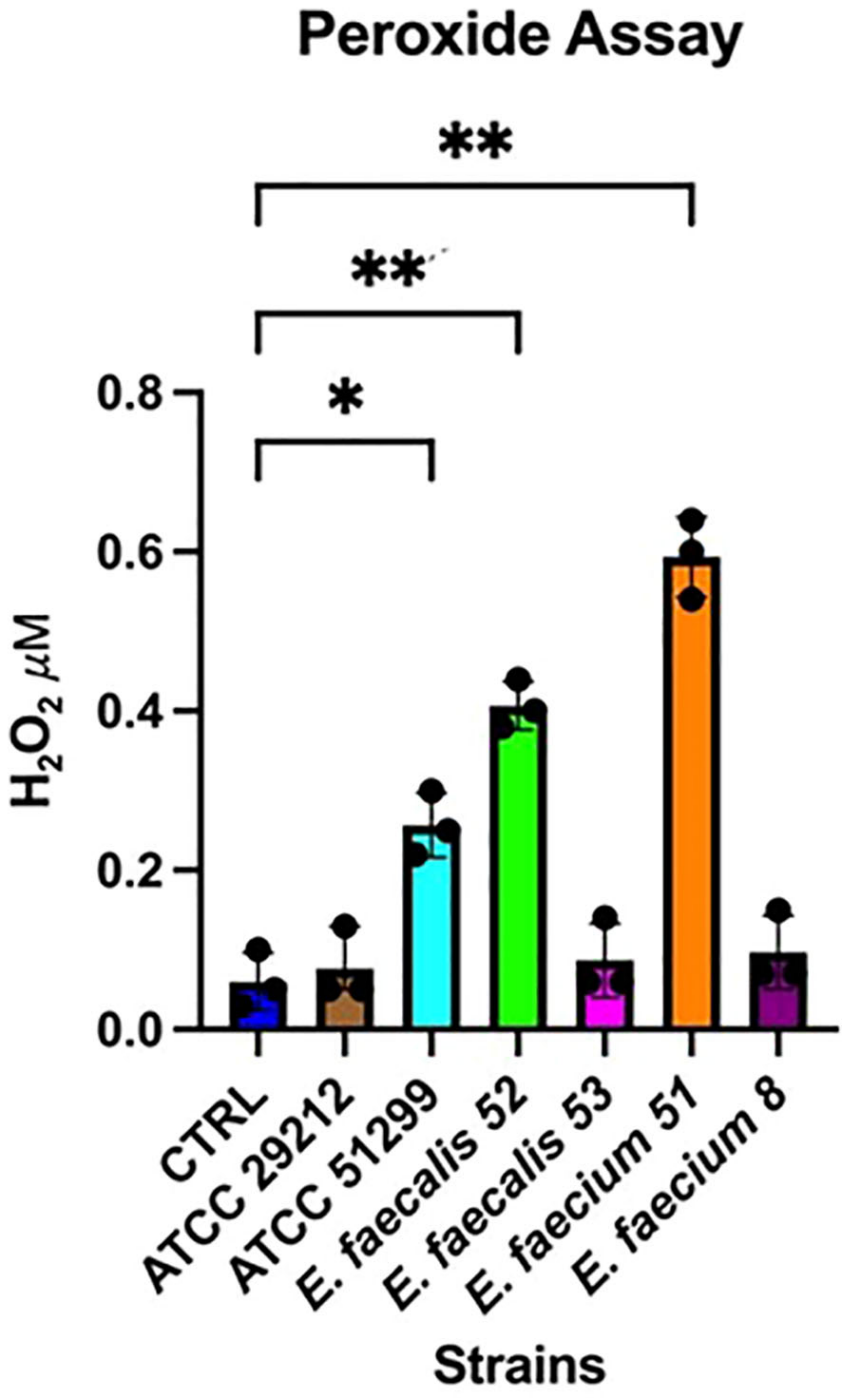
Induction of oxidative stress in Caco-2 cells following exposure to bacterial cell-free supernatants (CS). Each point represents an independent experiment (n = 3), each performed with five technical replicates. Error bars indicate the standard deviation (SD). Statistical analysis was performed using one-way ANOVA followed by Tukey's post hoc test; *p*<0.05 was considered statistically significant (*p<0.05; **p<0.01).

A comparable increase was also observed in cells treated with CS from the reference multidrug-resistant strain ATCC 51299™, which exhibited significantly higher levels of H_2_O_2_ release compared to controls (p < 0.05) (**Figure 6**). These results indicate that oxidative stress induction may represent a key mechanism underlying, at least in part, the cytotoxic effects of specific *Enterococcus* strains on the intestinal epithelium.

## Discussion

Our study provides complementary epidemiological, genomic, and functional insights into VRE clinical isolates from two Italian hospitals. The obtained results highlighted the complex host–pathogen interactions underlining possible dissemination episodes. Interestingly, the involved hospitals documented an increase in VRE isolation percentages among oncological patients during the study period. Certainly, this evidence especially regards only two healthcare settings, contributing to generalizability and limiting global considerations. Consequently, we collected only preliminary data to demonstrate the importance of investigating fragile patients’ characteristics and *Enterococcus* spp. colonization or infection. Despite a limited isolates number for the global microbiological and genomic analysis, our study showed interesting characteristics about resistome and virulome patterns of VRE isolates from different hospital settings. The cell models enriched our current knowledge about VRE adhesion capability. However, their performance should be increased, including more strains during future studies.

The demographic characteristics of our cohort reflected a predominantly immunocompromised and comorbid population, reporting a high prevalence of haematological malignancies. Microbiological data confirmed an *E. faecium* predominance, identifying the clonal complex 17 17-associated sequence types ST80 and ST117 for most strains. According to previous scientific data, these lineages are known for their adaptation to healthcare environments, extensive antibiotic resistance, and epidemic potential (Top et al., 2008; WHO, 2017; Organization WHO, 2022). The described extensive resistome confirmed our isolates’ multidrug-resistant phenotype and clinical relevance. The concurrent presence of virulence-associated genes supported the persistence and pathogenic potential of these clones in the nosocomial environment. The *E. faecalis* population displayed greater genetic diversity, spanning seven sequence types, with ST28 and ST179 emerging as the most represented lineages. These strains harbored classical virulence determinants such as *ebp* pili, capsule synthesis loci, and *gel*E, all of which have been implicated in urinary and bloodstream infections (Segawa et al., 2024; Zaheer et al., 2020; Dyrkell et al., 2021; Pratama et al., 2022).

Importantly, the host immune evasion modulator *elr*A and the cytolysin-encoding genes underscored the multifactorial nature of *E. faecalis* pathogenicity. These genomic findings highlight the convergence of resistance and virulence traits compromising high-risk patient populations (Rodríguez-Lucas et al., 2022). The high translocation capacity of *Enterococcus* spp. partially explains the elevated bacteremia incidence in immunocompromised patients. However, bacterial invasiveness alone does not account for dissemination (Sánchez-Díaz et al., 2016). Host–microbe interactions are multifaceted and influenced by external factors (e.g., cellular secretions, environmental conditions) as well as internal determinants (e.g., genotype, virulence factors) (Benson et al., 2010). Similar complex interactions may disrupt host physiology, but the specific impact of enterococcal secretory components on epithelial cells remains poorly defined. Moreover, clinical strains vary in their ability to directly interact with host cells through adhesion and colonization (Dunne et al., 2001).

Our *in vitro* assays provided functional evidence for strain-dependent interactions with the intestinal epithelium. Adhesion experiments showed that bloodstream-derived isolates adhered more strongly to Caco-2 cells than other clinical strains, suggesting a possible association between adhesive capacity and invasion. The robust adherence of *E. faecalis* ATCC 29212 validated adhesion as a key contributor to colonization and potential translocation. These findings supported the hypothesis that invasive isolates may exhibit enhanced adhesive phenotypes and facilitate gut barrier disruption for dissemination in fragile hosts (Long et al., 2019; Zhang et al., 2024). Beyond adhesion, several authors have hypothesized that secreted bacterial products may provide additional advantages by enhancing invasiveness. Previous studies have described the activity of enterococcal secreted components against host cells within *in vitro* and *in vivo* models (Castro et al., 2010; Alwehaibi et al., 2023). In our study, the cytotoxic effects observed following exposure to bacterial cell-free supernatants (CS) revealed an indirect mechanism of epithelial impairment.

The strongest reductions in Caco-2 viability were associated with bloodstream isolates (*E. faecium* 51 and *E. faecalis* 52) and the multidrug-resistant reference strain ATCC 51299. These results suggested that soluble bacterial products may exacerbate epithelial dysfunction beyond direct bacterial invasion. Previously published data regarding non-clinical strains similarly described antiproliferative activities of enterococcal supernatants, which were mainly attributed to proteinaceous metabolites (Sharma et al., 2018; Salek et al., 2023). In our study, we extended these observations to clinical multidrug-resistant isolates. Interestingly, the proposed analysis demonstrated that the oxidative stress may contribute to the cytotoxic activity of enterococcal secretomes. We observed significantly elevated H_2_O_2_ levels in epithelial cultures exposed to CS from invasive isolates and reference strains, with the highest outputs recorded for *E. faecium* 51, *E. faecalis* 52, and ATCC 51299. These data aligned with previous evidence that *Enterococcus* spp. generates reactive oxygen species as part of their metabolism, and genes such as *sod*A are implicated in oxidative stress regulation (Huycke et al., 2002; Lee et al., 2023). In addition, we investigated other genes involved in redox homeostasis, representing key components of the enzymatic machinery underlying the antioxidant defense of enterococci, including *npr*, *nox*, *ahp*C, *ahp*F, and *kat*A (Riboulet et al., 2007). Notably, small amino acid substitutions in *ahp*C can impair peroxiredoxin efficiency and lead to accumulation of H_2_O_2_, a mechanism potentially relevant to our isolates.

While we observed sequence variants in *nox* and *ahp*C, their predicted impact appears limited, although subtle alterations in the NADH oxidase – *ahpC/ahpF* redox cycle could influence peroxide turnover and oxidative balance (Moy et al., 2004; Toh et al., 2017). These observations supported a hypothetical link between redox-active components of the secretome and epithelial injury. Excessive H_2_O_2_ could plausibly contribute to the disruption of epithelial junctions, compromise barrier integrity, and facilitate bacterial translocation. These processes are particularly relevant in immunocompromised hosts (Jepson, 2003; Rao, 2008; Sangiorgio et al., 2024a; Charitos et al., 2025). However, our data remains correlative and does not prove a direct cause-and-effect relationship. Future studies could explore this further using antioxidant rescue assays, such as catalase treatment, to evaluate the potential role of H_2_O_2_ in epithelial stress (Boonanantanasarn et al., 2012). At the same time, other secreted molecules may act independently or in synergy with ROS. Indeed, comparative proteomic and metabolomic studies have shown that *Enterococcus* secretomes contain a variety of cytotoxic metabolites and proteins capable of modulating host cell viability (Alwehaibi et al., 2023; Salek et al., 2023). These findings broaden our understanding of VRE-epithelium interactions and are in line with earlier work on oxidative stress in *Enterococcus* pathogenesis, including Huycke et al. (2002, which highlighted the potential contribution of H_2_O_2_ to host cell injury (Jepson, 2003; Rao, 2008; Sangiorgio et al., 2024a; Charitos et al., 2025). According to our results, resistance and virulence mechanisms may act synergistically to promote bacterial translocation from the gut to the bloodstream (Prematunge et al., 2016; Papanicolaou et al., 2019; Poudel et al., 2023). Nevertheless, our study did not identify the specific secreted effectors involved, and additional candidates beyond metabolites warrant investigation. We hypothesized that other CS components, such as bacterial membrane vesicles, could contribute to the observed effects, consistent with their documented roles in host–pathogen interactions (Kim et al., 2019; Sangiorgio et al., 2024b; Leggio et al., 2025).

Future studies should incorporate metabolomic and proteomic approaches to characterize these factors and employ organoid or *in vivo* models to better mimic the intestinal microenvironment. Several limitations should also be acknowledged. The retrospective design limited access to longitudinal clinical data, and the *in vitro* analyses were restricted to a small subset of isolates and a single epithelial cell line, which may not fully recapitulate the complexity of the intestinal environment. In conclusion, our integrated clinical, genomic, and functional analyses demonstrate that VRE pathogenesis involves not only antibiotic resistance and classical virulence determinants but also the secretion of cytotoxic metabolites capable of inducing oxidative stress in the intestinal epithelium. These findings enhance our understanding of VRE biology and suggest that therapeutic strategies aimed at preserving epithelial integrity and counteracting oxidative damage may help mitigate invasive disease, particularly in high-risk patient populations.

## Materials and methods

### Data collection

This retrospective analysis included (VRE) isolates obtained over a five-month period (June 2023–October 2023) from two Italian hospitals: the University Hospital Policlinico of Catania, and the Civico-Di Cristina-Benfratelli Hospital of Palermo. Isolates were recovered either from routine microbiological surveillance specimens (e.g., rectal swabs, urine) or from diagnostic specimens collected as part of standard patient care (e.g., blood cultures, cerebrospinal fluid, biopsies, urine, and drainage fluids). No additional sampling was performed for the purposes of this study, and all clinical information was obtained retrospectively from medical records after complete anonymization. In line with these conditions, informed consent was not required by the local Research Ethics Committees. General clinical and demographic data were collected, including age, sex, site of isolation, laboratory parameters, comorbidities, recent surgical procedures, chemotherapy, use of immunosuppressive medications (e.g., corticosteroids), and prior antibiotic exposure. Microbiological data, such as species identification and antimicrobial susceptibility testing results, were recorded by clinical microbiology laboratory staff during the study period. The Charlson Comorbidity Index was applied to assess comorbidity burden and overall illness severity (Chuang et al., 2022).

### Strain identification and antimicrobial susceptibility testing

Species-level identification of *Enterococcus* isolates was performed in the clinical microbiology laboratories using matrix-assisted laser desorption/ionization time-of-flight mass spectrometry (MALDI-TOF MS) with the MALDI Biotyper^®^ Sirius System (Bruker, Billerica, MA, USA). Antimicrobial susceptibility testing (AST) was initially conducted with the VITEK^®^2 system and the VITEK^®^2 AST-P658 card (bioMérieux, Marcy l’Etoile, France), which provided minimum inhibitory concentrations (MICs) for amoxicillin/clavulanic acid, ampicillin, ampicillin/sulbactam, ciprofloxacin, levofloxacin, linezolid, quinupristin/dalfopristin, teicoplanin, tigecycline, and vancomycin. Results were interpreted according to the most recent EUCAST guidelines (The European Committee on Antimicrobial Susceptibility Testing, [[NoYear]]). All isolates were subsequently sent to the Medical Molecular Microbiology and Antimicrobial Resistance (MMAR) Laboratory, Department of Biomedical and Biotechnological Sciences, University of Catania, for confirmatory testing. MIC values were validated using the EUCAST reference broth microdilution method, which also included gentamicin susceptibility testing to assess for the presence of aminoglycoside-modifying enzymes. Each strain was tested in duplicate, yielding reproducible MIC values across replicates. *Enterococcus faecalis* ATCC 29212 was employed as the quality control strain for susceptibility assays.

### Molecular characterization

WGS was performed at the MMAR Laboratory to determine sequence type (ST) and to characterize the resistome and virulome of the isolates. Genomic DNA was extracted using the QIAamp^®^ DNA Mini Kit (Cat. No. 51304, QIAGEN, Hilden, Germany). DNA concentration and purity were assessed with an Eppendorf BioPhotometer^®^ D30 and a Qubit fluorometer, using the Qubit dsDNA HS Assay Kit (Cat. No. 32850, Invitrogen, Carlsbad, CA, USA). A total of 100 ng of DNA per sample was prepared for sequencing following the Illumina DNA Prep – (M) Tagmentation protocol (Cat. No. 20018707, Illumina Inc., San Diego, CA, USA). Indexing was performed with Nextera™ DNA CD Indexes (24 Indexes, 24 Samples; Cat. No. 20019105, Illumina). Library quality and concentration were evaluated using the Qubit dsDNA HS Assay Kit (Cat. No. Q32851, Invitrogen) and the Agilent^®^ High Sensitivity DNA Kit (Cat. No. 5067-4626).

Libraries were denatured and diluted according to the Illumina “Denature and Dilute Libraries Guide,” with a final loading concentration of 8.5 pM. Sequencing was carried out on the Illumina MiSeq platform using the MiSeq Reagent Kit v3 (Cat. No. 15043895, Illumina). Run setup and FASTQ generation were managed via the Local Run Manager v3 software (Illumina, 2018). Bioinformatic analysis of raw reads was conducted with the CLC Genomics Workbench (QIAGEN, Aarhus, Denmark) using the Microbial Genomics Module v22.0 (January 2022 release). This workflow enabled sequence typing, resistome profiling, and virulome characterization for all isolates. For all isolates, sequencing coverage and assembly-quality metrics were consistent with high-quality genome assemblies, with an average N50 value of approximately 51 kb, an average coverage depth of 90x, and a mean GC content of 37.58%.

### Bacterial strains culture

Following antimicrobial susceptibility profiling and genomic characterization, four representative *Enterococcus* strains (1 *E. faecium* ST80, 1 *E. faecium* ST117, 1 *E. faecalis* ST28, and 1 *E. faecalis* ST179) were randomly selected for *in vitro* assays. Specifically, we selected one random isolate for each major ST because ST80, ST117, ST28, and ST179 represented the most reported STs during the genomic characterization. Two pathogenic reference strains, *Enterococcus faecalis* ATCC 29212™ and ATCC 51299™, were also included. Strains were cultured on Bile Aesculin Agar (BHI) (Thermo Scientific™ Oxoid™, Cat. No. CM0888) and incubated overnight at 37 °C. After each use, isolates were preserved at –80°C in Tryptic Soy Broth (TSB) (Thermo Scientific™ Oxoid™, Cat. No. CM0129B) supplemented with 15% glycerol (Sigma-Aldrich, Cat. No. 49767). Growth kinetics of selected isolates were determined in supplemented DMEM (Cat. No. 11965092, Gibco) to mimic the Caco-2 culture environment. Growth curves were established by measuring optical density at 600 nm (Eppendorf BioPhotometer^®^) at multiple time points (0, 2, 4, 6, 8, 24, and 48 h). Dilutions were plated on Tryptic Soy Agar (TSA; Thermo Scientific™ Oxoid™, Cat. No. CM0131B) and incubated overnight at 37°C for colony enumeration.

### Eukaryotic cell culture

Human colorectal adenocarcinoma cells (Caco-2, HTB-37™, ATCC, Manassas, VA, USA) were used as the eukaryotic host model. Cells were maintained in 75 cm² flasks with high-glucose DMEM supplemented with HEPES (Cat. No. 11965092, Gibco), GlutaMAX™, (Cat. No. 35050061, Gibco), 10% fetal bovine serum (FBS; Cat. No. F7524, Sigma-Aldrich-Merck), and 100 U/mL penicillin/streptomycin (Cat. No. 15140148, Gibco). Cultures were incubated at 37 °C in a humidified 5% CO_2_ atmosphere, with medium renewed twice weekly.

### Adhesion assay

For adhesion assays, Caco-2 cells were seeded at a density of 5 × 10^5^ cells/well in 24-well plates (Corning, Life Science). After reaching confluence, monolayers were washed with PBS (Cat. No. 10010023 Gibco), switched to antibiotic- and serum-free DMEM, and infected with bacteria at a multiplicity of infection (MOI) of 1:100. After 2 h incubation (37 °C, 5% CO_2_), non-adherent bacteria were removed by PBS washes. Cells were lysed mechanically with a cell scraper, and lysates were plated on TSA for CFU enumeration. Adherence was expressed as a percentage of the initial inoculum to normalize for variations in bacterial input. Each experiment was independently repeated three times using separate bacterial cultures and cell passages, with each condition tested in triplicate wells.

### Preparation of bacterial cell-free supernatants and cytotoxicity assay

Bacterial cell-free supernatants (CS) were prepared by culturing isolates overnight in supplemented DMEM, adjusting inocula to 10^5^ CFU/mL, and incubating until the early exponential phase. Cultures were centrifuged at 800 × g for 10 min at 4 °C, and supernatants were filtered through 0.22 µm Primo Vacuum Filter Systems (Euroclone, Cat. No. EPVPE22250). DMEM processed identically without bacteria served as an additional control. The effects of bacterial CS on epithelial viability were evaluated using an MTT assay (Bivona et al., 2022). Caco-2 cells were seeded in 96-well plates (100 µL/well) and incubated at 37 °C for 24 h. Cells were then exposed to 200 µL of bacterial CS, control CS (bacteria-free DMEM), or left untreated (quality control). After 6 h and 24 h incubation, MTT reagent was applied, and absorbance was measured at 569 nm using a Synergy H1 multi-well plate reader (Biotek, Milan, Italy). Results were normalized to untreated controls and expressed as a percentage of cell viability. Each MTT assay was independently repeated three times, with each condition measured in three technical replicates.

### Hydrogen peroxide quantification assay

To investigate whether *Enterococcus* isolates produce metabolites capable of inducing oxidative stress in epithelial cells, we measured hydrogen peroxide (H_2_O_2_) release in Caco-2 cultures exposed to bacterial CS. H_2_O_2_ quantification was performed using the Amplex^®^ Red Hydrogen Peroxide/Peroxidase Assay Kit (Invitrogen, Thermo Fisher Scientific, USA Cat. No. A22188), following the manufacturer’s instructions with minor adaptations for epithelial cell assays. Fluorescence was measured with a Qubit™ fluorometer (Thermo Fisher Scientific), and H_2_O_2_ concentrations were determined using a standard curve generated from known H_2_O_2_ concentrations. Each condition was analyzed in quintuplicate across three independent experiments. Background fluorescence from the culture medium was subtracted from all readings.

### Statistical analysis

All quantitative data were analyzed using GraphPad Prism version 9.0 (GraphPad Software, San Diego, CA, USA). Clinical and epidemiological data were retrospectively collected from hospital records and reported as frequencies and percentages for categorical variables, or as means ± standard deviation (SD) or medians with interquartile ranges (IQR) for continuous variables. Comparisons among multiple groups were performed using one-way or two-way analysis of variance (ANOVA) with appropriate *post hoc* tests.

## Data Availability

The original contributions presented in the study are publicly available. These data can be found at http://www.ncbi.nlm.nih.gov/bioproject/1291476 (BioProject ID: PRJNA1291476).
