# Supplementary material for: Clinical, genomic, and functional characterization of vancomycin-resistant Enterococci from immunocompromised patients: insights into epithelial dysfunction and bloodstream infections
Source: Front Cell Infect Microbiol. 2025 Dec 16;15:1726074. doi: 10.3389/fcimb.2025.1726074 (PMC12748236; doi:10.3389/fcimb.2025.1726074)
Supplement: Supplementary file 1 [file Table1.docx]

**Supplementary Figure 1.** Clinical profiles of representative *Enterococcus* isolates used for *in vitro* assays.

| **ID** | **Age** | **Sex** | **Ward** | **Species** | **Source** | **Admission diagnosis** | **CCI^1^** |
| --- | --- | --- | --- | --- | --- | --- | --- |
| 52 | 82 | F | Neurology | *E. faecalis* | Blood | Brain Cancer | 5 |
| 53 | 48 | F | Hematology | *E. faecalis* | Stool | Acute Myeloid Leukemia | 4 |
| 51 | 74 | M | Internal Medicine | *E. faecium* | Blood | Pneumonia in Acute Myeloid Leukemia | 3 |
| 8 | 73 | F | Hematology | *E. faecium* | Stool | Non-Hodgkin's Lymphoma | 6 |

^1^ CCI: Charlson Comorbidity Index

**Supplementary Figure 2.** Microbiological profiles of representative *Enterococcus* isolates used for *in vitro* assays

| ID | MLST | AUG  S≤4 R>8 | AMP  S≤4 R>8 | AMS  S≤4 R>8 | QDA  S≤1 R>1 | CIP  S≤4 R>4 | LEV  S≤4 R>4 | LNZ  S≤4 R>4 | TEC  S≤2 R>2 | VA  S≤4 R>4 | TGC  S≤0,25 R>0,25 |
| --- | --- | --- | --- | --- | --- | --- | --- | --- | --- | --- | --- |
| 51 | ST117 | >16 | >16 | >16 | 1 | >4 | >4 | 1 | >16 | >16 | < 0,12 |
| 8 | ST80 | >16 | >16 | >16 | 1 | >4 | >4 | 1 | >16 | >16 | 0,5 |
| 53 | ST28 | < 2 | < 2 | < 2 | 1 | >4 | >4 | 2 | >16 | >16 | < 0,12 |
| 52 | ST179 | < 2 | < 2 | < 2 | 1 | >4 | >4 | 1 | >16 | >16 | < 0,12 |

Antibiotic tested: AUG, amoxicillin/clavulanic acid; AMP, ampicillin; AMS ampicillin/sulbactam; QDA, quinupristin/dalfopristin; CIP, ciprofloxacin; LEV, levofloxacin; LNZ, linezolid; TEC, teicoplanin; VA, vancomycin; TGC, tigecycline.
